# Immune Response to Invasive Group B *Streptococcus* Disease in Adults

**DOI:** 10.3201/eid2211.160914

**Published:** 2016-11

**Authors:** Morven S. Edwards, Marcia A. Rench, C. Daniela Rinaudo, Monica Fabbrini, Giovanna Tuscano, Giada Buffi, Erika Bartolini, Stefano Bonacci, Carol J. Baker, Immaculada Margarit

**Affiliations:** Baylor College of Medicine, Houston, Texas, USA (M.S. Edwards, M.A. Rench, C.J. Baker);; GSK Vaccines, S.r.l., Siena, Italy (C.D. Rinaudo, M. Fabbrini, G. Tuscano, G. Buffi, E. Bartolini, S. Bonacci, I. Margarit)

## Abstract

Immunization of nonpregnant adults could help prevent invasive group B *Streptococcus* (GBS) infections, but adult immune responses have not been investigated. We defined capsular polysaccharide (CPS) and pilus island (PI) surface antigen distribution and expression and immune responses to GBS infection in nonpregnant adults. Prospective surveillance from 7 hospitals in Houston, Texas, USA, identified 102 adults with GBS bacteremia; 43% had skin/soft tissue infection, 16% bacteremia without focus, and 12% osteomyelitis. CPS-specific IgG was determined by ELISA and pilus-specific IgG by multiplex immunoassay. CPS types were Ia (24.5%), Ib (12.7%), II (9.8%), III (16.7%), IV (13.7%), and V (12.7%); 9.8% were nontypeable by serologic methods. Pili, expressed by 89%, were most often PI-2a. CPS and pilus-specific IgG increased during convalescence among patients with strains expressing CPS or PI. All GBS expressed CPS or PI; 79% expressed both. Increased antibodies to CPS and PI during recovery suggests that GBS bacteremia in adults is potentially vaccine preventable.

## Abstract 2

Antibodies to capsular polysaccharides and pilus proteins develop in recovering adults.

In the United States, group B *Streptococcus* (GBS) has emerged as a frequent cause of invasive infection in nonpregnant adults with underlying medical conditions. The incidence of GBS disease among these patients increased from 3.6 cases/100,000 persons in 1990 to 7.3 cases/100,000 persons in 2007 (p<0.001) (*1*). This increase has occurred among adults 18–64 years of age and >65 years of age (*2*). The Centers for Disease Control and Prevention Active Bacterial Core surveillance estimated that 28,000 cases of invasive GBS disease occurred in the United States during 2014 and that 25,900 (93%) cases occurred beyond infancy, primarily in nonpregnant adults (*3*). Also, adults account for 90% of the estimated 1,660 annual deaths attributable to GBS infection.

Most cases of invasive GBS disease are caused by 5 capsular types (Ia, Ib, II, III, and V), which together accounted for 88% of cases in nonpregnant adults during 1999–2005 (*2*). The emergence of serotype V in the 1990s was first noted among GBS isolates from nonpregnant adults (*4*,*5*). During 1999–2005, infection with the formerly rare serotype IV increased among nonpregnant adults (*1*). As with invasive pneumococcal disease, the capsule of GBS is a major virulence factor targeted by candidate GBS capsular polysaccharide (CPS)–protein conjugate vaccines; thus, knowledge of prevalence of these antigens is critical for vaccine development. Similarly, another surface component of GBS critical to virulence, the pilus islands (PIs), are limited in number but are found in all strains and are promising as GBS vaccine candidates (*6*–*8*).

Among infants, use of antenatal screening and intrapartum antimicrobial prophylaxis has markedly reduced early-onset (0–6 days of age) but not late-onset (7–89 days of age) GBS disease. Immunization of pregnant women offers the best strategy for prevention of early- and late-onset invasive GBS disease in infants. Immunization of adults also potentially could reduce the GBS disease burden in the United States, but data are needed regarding immune responses during convalescence from invasive disease (*9*). We characterized CPS and PI genotypes and expression among isolates and explored immune responses to these surface antigens during convalescence from invasive infection in a cohort of nonpregnant adults.

## Methods

### Participants and Setting

We identified cases of GBS bacteremia by conducting prospective, laboratory-based surveillance from March 2012 through October 2014 at 4 Texas Medical Center hospitals (Houston, TX, USA) and 3 suburban affiliates of 1 Texas Medical Center hospital, all of which provided uniform diagnostic evaluations and medical care. Invasive infection was defined as isolation of GBS from blood or cerebrospinal fluid. Potential cases were identified daily at participating hospital laboratories. Nonpregnant adults >18 years of age with invasive GBS infection were eligible for enrollment. We excluded those with symptom duration >7 days, HIV infection, or polymicrobial bacteremia. An investigator contacted the physician of record for approval before approaching the patient. Study personnel then discussed the study and eligibility with patients and obtained informed consent. The study received approval by the Institutional Review Boards for Human Research at Baylor College of Medicine and participating hospitals’ administrative processes.

### Specimen Collection

A blood sample was collected from each participant on the day of hospital admission or at enrollment. Demographic and clinical data were recorded from the medical record. Convalescent-phase blood was collected at a mean of 3 (range 2–4) weeks from the date of positive culture. Blood was transported to the investigators’ laboratory (Baylor College of Medicine, Houston, TX, USA) where serum was separated, aliquoted, and stored at −80°C until sent to GSK Vaccines (Siena, Italy) for testing.

### Bacterial Strains, Media, and Growth Conditions

GBS isolates from each patient were obtained from hospital laboratories. They were then grown in Todd Hewitt Broth (Becton Dickinson, Franklin Lakes, NJ, USA) at 35°C–37°C for serotyping and inoculated directly from plates into trypticase soy broth with 20% glycerol for storage at −80°C.

### Serotyping of GBS Isolates

Capsular typing was performed for all isolates. Both the capillary precipitin method (*10*) and the Strep-B-Latex rapid agglutination method (Statens Serum Institut, Copenhagen, Denmark) were used.

### Genotyping and Pilus Typing of GBS Isolates

Genomic DNA was prepared from GBS cultures by a standard protocol for gram-positive bacteria by mutanolysin treatment of bacteria and use of a GenElute Bacterial Genomic DNA Kit (Sigma-Aldrich, Milan, Italy) according to the manufacturer’s instructions. Capsular gene typing was performed for all isolates by multiplex PCR (*11*) modified by including a primer pair specific for detection of serotype IX. Pilus genes were PCR amplified by using primers specifically annealed to conserved genomic regions external to the coding sequences as described (*8*). 

### Multilocus Sequence Typing 

Multilocus sequence typing was performed as described (http://pubmlst.org/sagalactiae/). Clonal complexes (CCs) were assigned by global optimal eBURST analysis of multilocus typing data (*12*).

### Fluorescence-Activated Cell Sorter Analysis

Fluorescence-activated cell sorter (FACS) analysis to assess surface expression of CPS and pilus proteins was performed as reported (*13*,*14*). In brief, paraformaldehyde-fixed bacteria were incubated with mouse monoclonal antibodies or immune polyclonal serum specific for CPS type and pilus proteins (*14*). Cells were stained with R-phycoerythrin-conjugated F(ab)_2_ goat anti-mouse IgG (Jackson ImmunoResearch Laboratories, West Grove, PA, USA) and analyzed with a FACS CANTO II instrument (Becton Dickinson) and FlowJo Software (Tree Star Inc., Ashland, OR, USA).

### ELISA

Serum CPS-specific IgG was determined by ELISA. Microtiter plates (96-well) (Nunc MaxiSorp; Sigma Aldrich, St. Louis, MO, USA) were coated overnight at 2°C–8°C with 100 ng of purified CPS Ia, Ib, II, III, IV, or V conjugated to human serum albumin-adipic acid dihydrazide in phosphate-buffered saline (PBS) at pH 7.4. After washing with 0.05% Tween-20 in PBS (PBST), plates were incubated for 90 min with 2% bovine serum albumin in PBST. Serial 2-fold dilutions of test and standard serum in 2% bovine serum albumin in PBST were added, incubated 1 h, and washed. After alkaline phosphatase-conjugated anti-mouse IgG (1:1000 in PBST) was added, plates were incubated for 90 min and then washed. After incubation with p-nitrophenylphosphate (4.0 mg/mL), the reaction was stopped with EDTA 7% (wt/vol) and absorbance was measured at 405 nm. The concentration of specific IgG was determined with a standard titration curve and expressed as micrograms per milliliter (CPS Ia, Ib, III, and V) or ELISA units per milliliter (CPS IV). Standard samples containing 47.2, 35.4, 30.4, 83.5, or 11.6 μg/mL of anti-CPS Ia, Ib, II, III, and V IgG were prepared by pooling serum from women immunized with the specific CPS conjugated to tetanus toxoid (*15*). The CPS IV standard was prepared by pooling unweighted high-titer serum from study participants. The lower limit of quantitation was 0.156 μg/mL for types Ia, Ib, and V; 0.250 μg/mL for types II and III; and 12.5 ELISA units/mL for type IV. A titer corresponding to one half of the lower limit of quantitation was assigned to serum with values below the lower limit of quantitation.

### Multiplex Immunoassay 

Pilus protein–specific IgG was measured by multiplex immunoassay that used recombinant pilus proteins BP-1, BP-2a, and BP-2b coupled to magnetic beads and expressed as relative Luminex units (RLU) per milliliter. A total of 20 μg of each protein was coupled to carboxyl groups of a specific MagPlex bead set (Luminex Corporation, Austin, TX, USA). Three sets of coupled beads (3,000 beads/region/well) were added to each dilution of the standard curve and serum samples (1:100 and 6 serial 2-fold dilutions), incubated for 90 min in the dark, with shaking at 700 rpm. Beads were washed twice with 200 μL of PBS and incubated with phycoerythrin-conjugated goat anti-human IgG F(ab′)_2_ (Jackson ImmunoResearch Laboratories) at 5 μg/mL for 1 h in the dark, with shaking at 700 rpm. After a second wash, beads were suspended in 100 μL PBS. 

Analysis was performed on a FlexMAP3D instrument by using Bio-Plex Manager software version 6.0 (Bio-Rad, Hercules, CA, USA). For each analyte, median fluorescent intensity was converted to RLU per milliliter by interpolation from a 5-parameter logistic standard curve for every bead-region/standard. Standard serum for determination of human IgG specific for GBS pilus proteins was obtained by pooling hyperimmune serum from persons colonized by GBS. A titer of 1 RLU/mL was arbitrarily assigned to the highest concentration point of the calibration curve. The lower limit of quantitation for the pilus proteins was 0.14 RLU/mL for BP-1 and 0.41 RLU/mL for BP-2a and BP-2b.

### Statistical Analyses

Clinical features, by patient age group, were compared by using the 2-tailed Fisher exact test. Antibody responses to CPS or pilus proteins were assessed by paired *t*-test on log-transformed data. CPS-specific IgG concentrations were compared for homologous and heterologous (Mann–Whitney U test) GBS serotypes.

## Results

### Participant Characteristics

Participants comprised 102 nonpregnant adults (race/ethnicity 44% white, 30% black, 25% Hispanic, 1% other) with GBS bacteremia. Mean age was 59.5 years (median 59.7, range 27–91 years). Most (75%) were from Texas Medical Center hospitals. Most (59%) were male. One patient also had meningitis. All patients had >1 condition that enhanced risk for invasive GBS disease, including diabetes mellitus (59%), obesity (57%), cardiovascular disease (45%), liver disease (20%), kidney disease (19%), or cancer (10%) (*1*,*2*). The most common expressions of illness were skin/soft tissue infection (43%) bacteremia without focus of infection (16%), osteomyelitis (12%), and endocarditis or endovascular infection (8%). Four patients died, 2 of them before convalescent-phase serum was obtained.

### Characteristics of Infecting GBS Isolates

**Figure 1:**
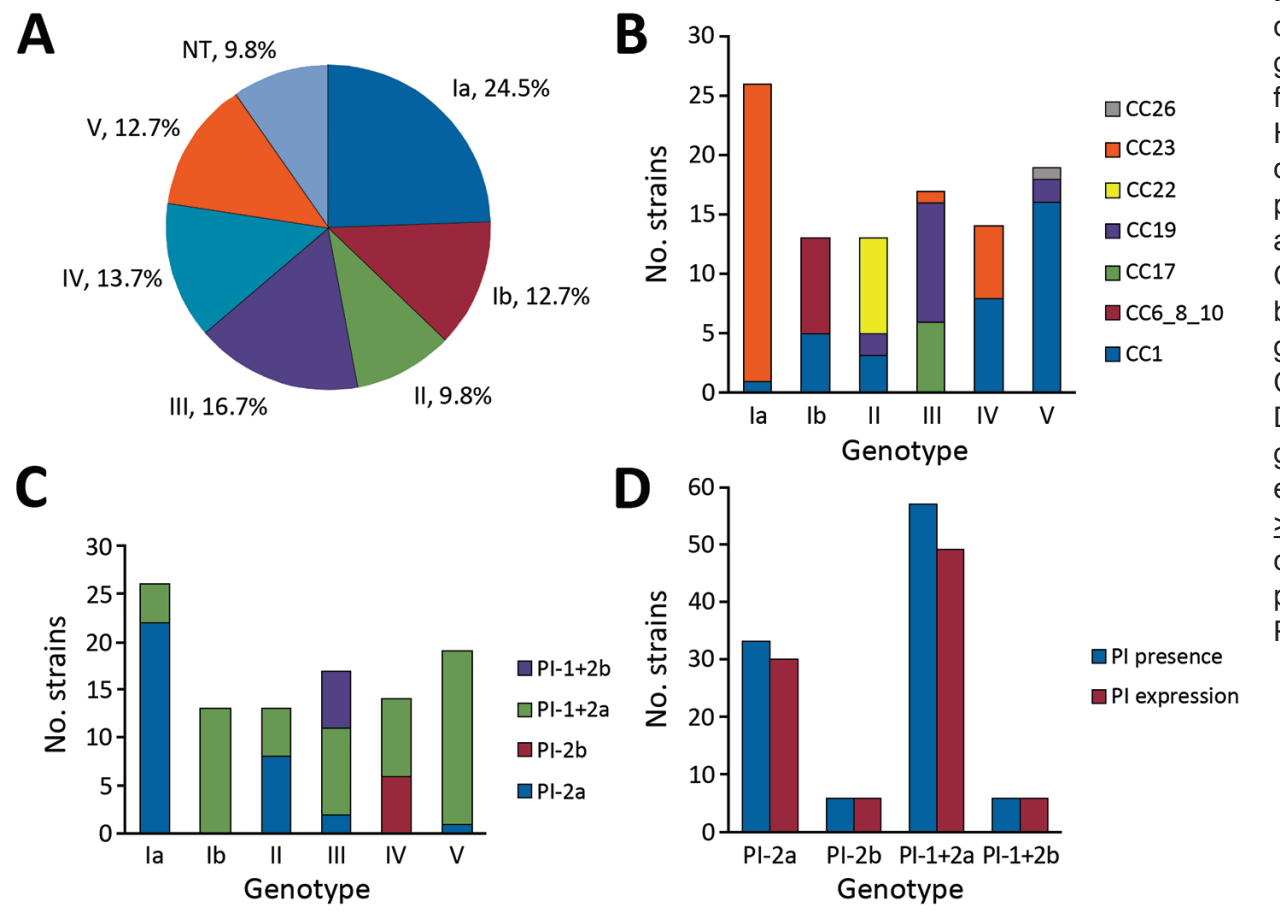
CPS type and PI distribution and expression in 102 group B *Streptococcus* isolates from adults with invasive infection, Houston, Texas, USA. A) CPS distribution of isolates by capillary precipitin method and latex agglutination assay (indicating CPS expression). B) Correlation between CC distribution and CPS genotype by PCR. C) PI and CPS distribution by genotype. D**)** Relationship between pilus genotype distribution and pilus expression in isolates expressing >1 pilus type on their surface. CC, clonal complex; CPS, capsular polysaccharide; NT, nontypeable; PI, pilus island.

Of 102 isolates, 92 expressed 1 of 6 capsular types. CPS type Ia accounted for 24.5% of isolates, followed by types III (16.7%), IV (13.7%), V (12.7%), Ib (12.7%), and II (9.8%) (Figure 1, panel A). The genotype and serotype for these 92 isolates were concordant. CPS expression was undetectable by latex agglutination and capillary precipitin methods for 10 (9.8%) isolates; 7 were identified by FACS analysis as type Ia (1), II (3), or V (3). The genotypes for the 10 isolates were Ia (1), II (3), and V (6). Genotypes and CCs for all 102 isolates are displayed (Figure 1, panel B); 77% of these isolates were 1 of the 3 lineages (CC1, CC19, or CC23). The hypervirulent CC (CC17) accounted for 35% of type III isolates. Among type IV isolates, CC1 (57%) and CC23 (43%) were exclusively represented.

PI genes were detected in all isolates (Figure 1, panels C and D). Most carried genes for PI-1 and PI-2a (56%) or PI-2a alone (32%). According to flow cytometry, 89% of isolates expressed in vitro >1 pilus type on their surface, most often PI-2a alone or with PI-1. PI-2b was expressed only by type IV strains or in combination with PI-1 by type III strains. Genes for PI-1 and PI-2a were present on 8 of 11 isolates not expressing pili. The CPS types for the 11 strains that did not express pili were Ia (1), Ib (2), II (4), IV (2), and V (2). All GBS strains expressed either CPS or pili, and 79% expressed both surface antigens.

### Immune Responses 

**Figure 2:**
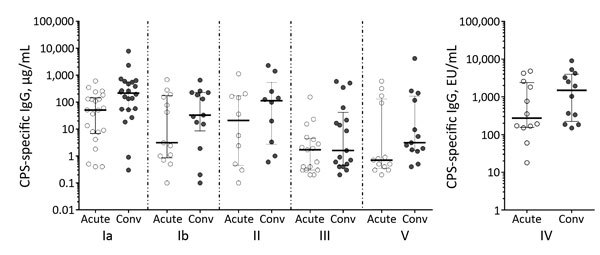
CPS-specific IgG concentrations/titers in acute- versus convalescent-phase serum from patients with group B streptococcal infection, Houston, Texas, USA. Horizontal bars represent median concentrations (± interquartile range) for each patient group. Conv, convalescent; CPS, capsular polysaccharide; EU, ELISA units.

Paired acute- and convalescent-phase serum samples were available from 97 patients, but only 87 were infected with CPS-expressing strains. A significant increase in CPS-specific IgG was observed during the convalescent phase for each of the 6 GBS types causing invasive disease in these 87 patients (Table). However, the concentration of antibodies to CPS in acute- and convalescent-phase serum varied widely (Figure 2). When CPS-specific IgG was expressed as >4-fold increases during convalescence, an immune response to their infecting serotype was detected for 50% (Ia), 31% (Ib), 50% (II), 41% (III), 42% (IV), and 46% (V) of patients.

**Table:** CPS-specific IgG responses for 87 nonpregnant adults with invasive GBS infection*

GBS type	CPS-specific IgG, GMC (range) [95% CI], μg/mL				
	No. patients†	Acute-phase sample		Convalescent-phase sample	p value‡
Ia	22	26.0 (0.39–609.1) [9.5–71.0]		137.6 (0.35–7,792.2) [50.4–375.6]	<0.001
Ib	13	9.3 (0.08–680.9) [1.6–55.6]		25.2 (0.08–650.5) [4.6–139.5]	0.035
II	10	11.6 (0.13–1,126.8) [1.2–113.6]		47.4 (0.61–2,279.9) [6.1–366.1]	0.032
III	17	1.5 (0.13–153.9) [0.53–4.0]	4.8 (0.13–584.4) [1.1–20.4]		0.044
V	13	3.2 (0.24–596.3) [0.51–19.9]	10.1 (0.43–4,182.0) [1.9–55.0]		0.003
IV§	12	409.5 (17.6–4,823.3) [133.3–1,257.5]	1,060.9 (149.4–9,085.2) [415.7–2,707.6]		0.022

*CPS, capsular polysaccharide; GBS, group B *Streptococcus*; GMC, geometric mean concentration. †Excludes 10 patients with infection caused by GBS that did not express capsular polysaccharide, 3 for whom convalescent-phase samples were of insufficient quantity for analysis, and 2 who died before collection of convalescent-phase serum. ‡Paired *t*-test on log-transformed data. §Results are expressed in ELISA units/mL.

**Figure 3:**
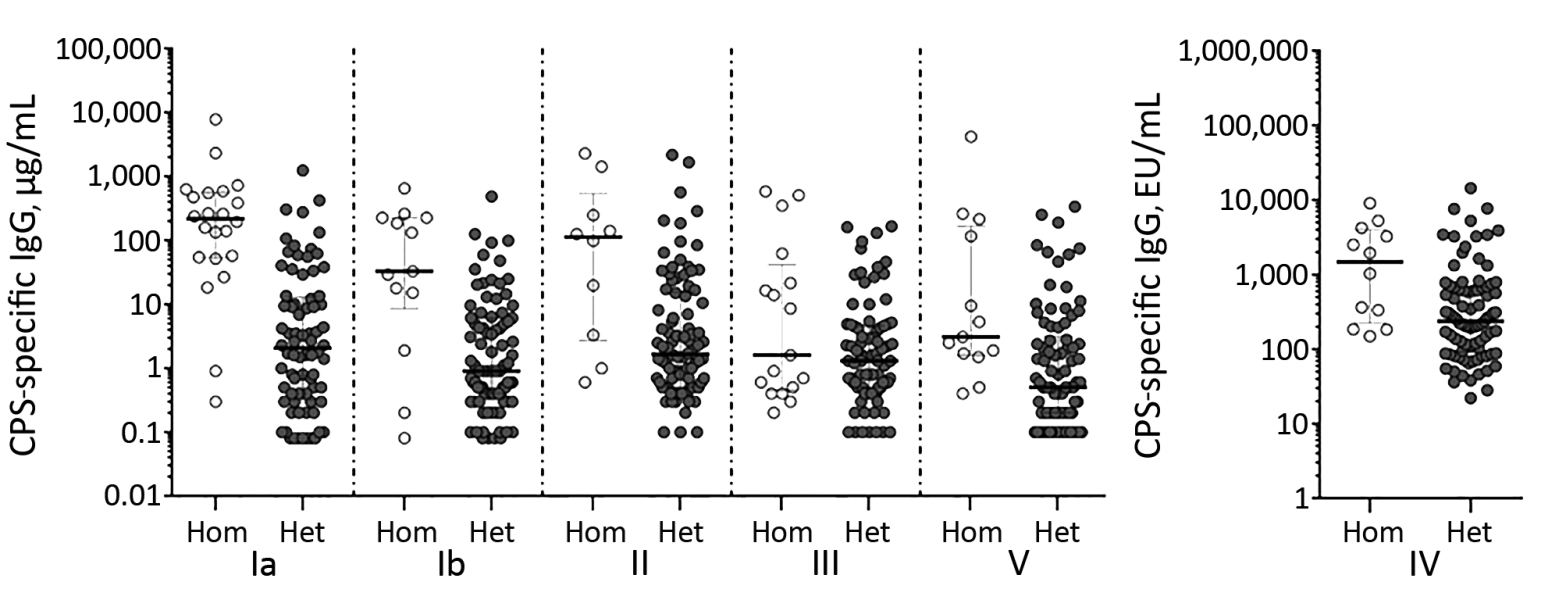
Concentrations of CPS-specific IgG against homologous or heterologous group B streptococcal serotypes in convalescent-phase serum samples from infected patients, Houston, Texas, USA. Horizontal bars indicate median concentrations (± interquartile range) within each group. CPS, capsular polysaccharide; EU, ELISA units; het, heterologous; hom, homologous.

For all patients, CPS-specific IgG concentrations in convalescent-phase serum samples were compared with the infecting GBS serotype and with heterologous GBS serotypes (Figure 3). With the exception of type III, the means and interquartile ranges of CPS-specific IgG were significantly higher (p<0.001) for the infecting GBS serotype than for the other 5 serotypes, indicating that the CPS immune response in adults is capsular type specific.

**Figure 4:**
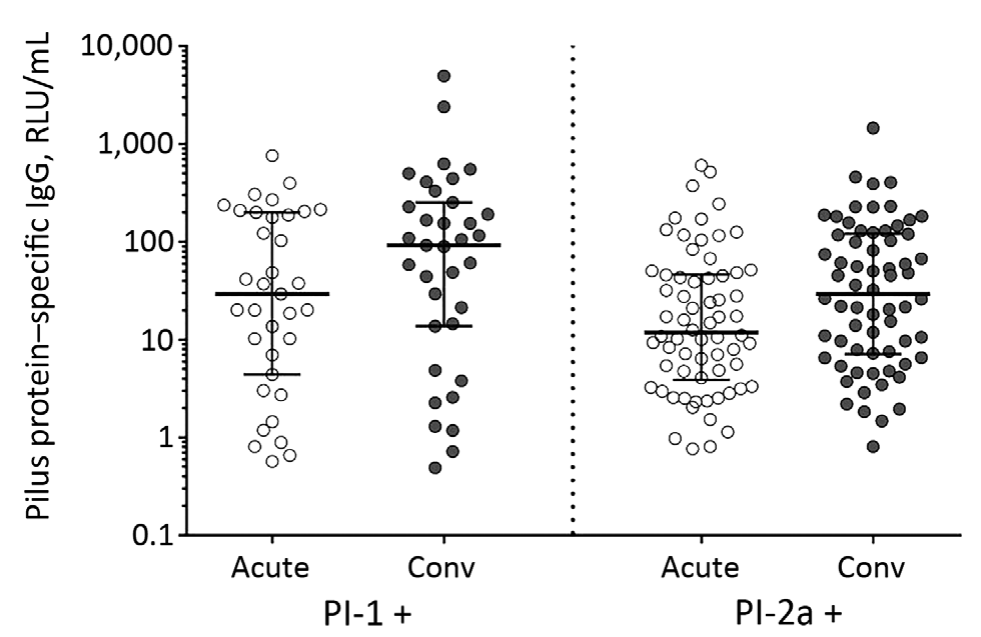
Pilus-specific antibody responses in acute- and convalescent-phase serum from patients infected with group B streptococcal strains expressing pilus type 1 or type 2a, Houston, Texas, USA. Anti-pilus–specific IgG titers were measured by multiplex immunoassay that used recombinant pilus proteins coupled to magnetic beads and expressed in RLU/mL. Horizonal bars represent the median (± interquartile range) within each population. For both comparisons, anti-pilus IgG increased significantly in serum collected during the acute and convalescent phases (p<0.001). p values were calculated by *t*-test on log-transformed data. Conv, convalescent; RLU, relative Luminex units.

PI-1–specific IgG increased significantly for the 35 patients infected with GBS strains expressing PI-1 when acute-phase serum (geometric mean concentration [GMC] 25.2 RLU/mL [range 0.57–765.0; 95% CI 12.2–52.0]) was compared with convalescent-phase serum (GMC 53.7 RLU/mL [range 0.5–4972.1; 95% CI 24.3–118.7]) (p = 0.003). Similarly, PI-2a–specific IgG increased significantly between acute illness (GMC 15.0 RLU/mL [range 0.8–606.5; 95% CI 10–22.5]) and convalescence (GMC 28.7 RLU/mL [range 0.8–1459.9; 95% CI 19.43.2]) for the 66 patients with infection caused by GBS isolates expressing this pilus type (p<0.001) (Figure 4). Among the 12 patients infected with GBS strains expressing PI-2b, there was no significant PI-2b–specific IgG response (data not shown). We detected >4-fold increases in pilus-specific IgG during convalescence in 20% (PI-1), 16.7% (PI-2a), and 25% (PI-2b) of patients. Among these 20 patients, response to their infecting CPS type was also >4-fold for 14 (70%).

## Discussion

GBS is a common pathogen in nonpregnant adults (*16*). Skoff et al. (*1*) suggest that the increasing prevalence of chronic medical conditions might explain the increasing incidence of GBS disease among adults. Most patients in our cohort had underlying conditions, as has been noted previously (*1*,*17*–*19*). Skin/soft tissue infections were present in 43%, and the presentation was nonfocal for 16%, usually in patients with hepatic disease (*1*,*2*).

Our investigation led to 3 major findings. First, serotype IV GBS was a prominent cause of invasive disease, accounting for 13.7% of cases. Skoff et al. (*1*) noted that type IV cases increased from 0.2% during 1998–1999 to 5.7% during 2005–2006. Characterization of 549 strains from Canada during 2010–2014 indicated that 16.9% of cases with invasive disease were caused by serotype IV (*20*). In our cohort, 6 GBS types were responsible for at least 10% of cases, but only types Ia (24.5%) and III (16.7%) caused more infections than type IV. None of the cases reported here were caused by GBS types VI–IX; Skoff et al. (*1*) identified only 3 of these types among 1,933 isolates from nonpregnant adults. Invasive type IV GBS infections were clinically diverse and included bacteremia without focus (4 patients), skin and soft tissue infections (3), osteomyelitis (3), septic shock (1), endocarditis (1), arthritis (1), and urosepsis (1).

Second, our findings add to limited data regarding GBS virulence factors critical to invasive disease pathogenesis in adults. Most GBS isolates can be assigned to a small number of CCs, including CC1, CC10, CC17, CC19, and CC23 (*21*). Three major lineages (CC1, CC19, and CC23) accounted for 77% of isolates from our cohort. These 3 CCs comprised 64% of isolates from nonpregnant adults in Sweden who had invasive infections during 1988–1997 (*22*). CC19, associated with less invasive type III strains in neonates, accounted for 63% of type III isolates in the patients reported here; the hypervirulent CC17 comprised only 33%, suggesting that less virulent strains may cause invasive disease in adults (*23*,*24*). Among type IV isolates, CC1 and CC23 were exclusively represented, extending the observations of Ferrieri et al (*25*) that type IV isolates from nonpregnant adults with invasive disease were CC1 and that those from invasive infection in infants were CC23. No type IV isolates belonged to the novel epidemic hypervirulent CC17 lineage resulting from the type III to type IV capsular switch (*26*,*27*). Teatero et al. (*20*) assert that emergence of serotype IV in Canada was driven by CC1 sequence type 459 strains, possibly linked to acquisition of resistance to tetracycline, macrolides, and lincosamides.

All strains isolated from the patients reported here expressed CPS, pili, or both surface-associated GBS virulence factors. For a subset of strains, expression was undetectable for CPS (10%) or pilus (11%). The proportion of nontypeable strains (assessed by conventional CPS typing methods) in our cohort was greater than the 6.5% reported by the Centers for Disease Control and Prevention during 2005–2006 (*1*). Also, the proportion of strains for which pilus expression was undetectable was slightly higher than the 6% reported for 289 GBS isolates from the United States and Italy, but these data represented infants and adults with either invasive infection or asymptomatic colonization (*8*).

Third, as a group, nonpregnant adults demonstrated an immune response to major virulence antigens after invasive GBS infection. CPS-specific IgG to each infecting CPS type and PI-1– or PI-2a–specific IgG almost always increased significantly in convalescent-phase serum. However, the immune response was complex; the range of antibody concentrations in acute- and convalescent-phase serum was broad, a pattern not found in neonates and infants, for whom low concentrations of CPS-specific IgG are uniformly found in acute-phase serum (*28*).

We observed several patterns of immune response. Between acute illness and convalescence, CPS-specific IgG increased by >4-fold for 44% of patients. By comparison, CPS-specific IgG increased by >4-fold in postimmunization serum from 80%–93% of healthy adults immunized with monovalent GBS conjugate vaccines representing CPS Ia, Ib, II, III, and V (*29*). For other patients, CPS-specific IgG in concentrations at the time of hospital admission were higher than the 0.5–3 μg/mL predicted to be protective against neonatal infection (*30*), suggesting that either the immune response was rapid or that an immune response to CPS is not as critical for nonpregnant adults with invasive infection as it is for neonates. Wessels et al. (*31*) noted that the serum CPS-specific IgG to the infecting GBS isolate in adults with bacteremia was >3.5 μg/mL in 7 of 12 serum samples obtained within 2 days of admission. Although acute-phase serum from the patients reported here was almost always collected at the time of admission and those who had symptoms >7 days before admission were excluded from enrollment, for some, a rapid, anamnestic response could have developed before admission. Many patients had diabetes and lower extremity infection or chronic lower extremity disease that could have led to prolonged antigenic stimulation resulting in a high level of CPS-specific IgG that declined during convalescence.

Our study has some limitations. Because we enrolled patients from only 1 city, the diversity of surface antigens relevant to immune response might not be applicable to other regions. Despite obtaining acute-phase serum at admission, the time of bacteremia onset could not be determined, complicating interpretation of immune response results. Our study was not designed to evaluate functional aspects of immune responses, which will be relevant to vaccine design considerations.

One requirement for a robust protective response is surface availability of antigens for antibody recognition (*7*). In murine models of GBS disease, pilus components induce protective immunity against all tested GBS challenge strains (*8*), and sufficiently high CPS-specific antibodies are associated with disease protection for newborn infants (*30*,*32*). The concept that CPS-protein conjugate vaccines are immunogenic in older adults also is well established (*33*). A 13-valent polysaccharide conjugate vaccine has proven effective at preventing vaccine-type invasive disease caused by *Streptococcus pneumoniae*, another pathogen that results in serious illness in adults with underlying medical conditions and those >65 years of age (*34*). Theoretically, a GBS conjugate CPS vaccine also incorporating pilus protein surface antigens could elicit a protective immune response providing protection for those adults at risk for invasive infection.
